# Roles and action mechanisms of WNT4 in cell differentiation and human diseases: a review

**DOI:** 10.1038/s41420-021-00668-w

**Published:** 2021-10-12

**Authors:** Quanlong Zhang, Yan Pan, Jingjing Ji, Yuxin Xu, Qiaoyan Zhang, Luping Qin

**Affiliations:** College of Pharmaceutical Science, Zhejiang Chinese Medical University, 311402 Hangzhou, China

**Keywords:** Predictive markers, Prognostic markers

## Abstract

WNT family member 4 (WNT4), which belongs to the conserved WNT protein family, plays an important role in the development and differentiation of many cell types during the embryonic development and adult homeostasis. Increasing evidence has shown that WNT4 is a special ligand that not only activates the β-catenin independent pathway but also acts on β-catenin signaling based on different cellular processes. This article is a summary of the current knowledge about the expression, regulation, and function of WNT4 ligands and their signal pathways in cell differentiation and human disease processes. WNT4 is a promoter in osteogenic differentiation in bone marrow stromal cells (BMSCs) by participating in bone homeostasis regulation in osteoporotic diseases. Non-canonical WNT4 signaling is necessary for metabolic maturation of pancreatic β-cell. WNT4 is also necessary for decidual cell differentiation and decidualization, which plays an important role in preeclampsia. WNT4 promotes neuronal differentiation of neural stem cell and dendritic cell (DC) into conventional type 1 DC (cDC1). Besides, WNT4 mediates myofibroblast differentiation in the skin, kidney, lung, and liver during scarring or fibrosis. On the negative side, WNT4 is highly expressed in cancer tissues, playing a pro-carcinogenic role in many cancer types. This review provides an overview of the progress in elucidating the role of WNT4 signaling pathway components in cell differentiation in adults, which may provide useful clues for the diagnosis, prevention, and therapy of human diseases.

## Facts

- WNT4 plays a promoter in osteogenic differentiation, β-cell maturation, decidual cell differentiation, neuronal differentiation, dendritic cell differentiation, and myofibroblast differentiation as well as a pro-carcinogenic role in cancer cells.
- The upstream regulators of WNT4 include transcription factor EGR1, PAX2, and FOXC2, which can promote transcription of WNT4 by binding to its promoter.
- WNT4 protein activates both β-catenin-dependent and β-catenin-independent pathways based on different cellular processes.

## Open questions

- What are regulative mechanisms of WNT4 in different cells during the process of human diseases?
- What is the function of WNT4 in human diseases?
- How can we aggregate the research achievement of WNT4 protein into the human disease treatments?

## Introduction

The WNT family contains 19 WNT genes, which work as key regulators in many physiological processes such as body growth and development, stem cell differentiation and cancer development by encoding secreted lipoglycoproteins [1]. The 19 WNT family proteins are categorized into canonical ligands (Wnt1 and Wnt3a) and non-canonical ligands (Wnt5a, Wnt4, Wnt11, and Wnt7a) based on their dependence on transduction through β-catenin. WNT4 is one of the most extensively studied prototypical ligands of the Wnt family and is known to play a key role in the developmental process of various organs and cells. A previous review reports that WNT4 dysregulation during the embryonic period may result in male-to-female sex reversal [2], which is critical in the development of sexual organs and mammary glands [3], while a decreased expression of WNT4 polymorphism is associated with reduced bone density with premature skeletal aging [4], endometriosis in infertile patients [5], and gynecological malignancies [6]. Despite these observed critical roles of WNT4 in both normal and malignant tissues, WNT4 signaling is crudely understood due to varied context-dependent functions. WNT4 has been shown to regulate either β-catenin-dependent or β-catenin-independent signaling and can either activate or suppress signaling (described in references herein). As such, WNT4 has been described as a “problem child” among WNT proteins. So, one of the goals of WNT4 study is to clarify the signaling pathway of WNT4. In addition, recent reports suggest that the abnormality of WNT4 signaling is involved in various cancers [7] and ovary [8], kidney [9], bone, and metabolic disorders [10]. However, there is little knowledge about the whole picture of WNT4 signaling, its activity, and WNT4-related diseases at present. In this review, we first provide an overview of the properties of WNT4 itself and then describe its upstream regulators and downstream signaling pathways. Then we summarize the role of WNT4 in differentiation of different cell types. Finally, we discuss the expression and function of WNT4 signaling in human diseases, including cancer, tissue fibrosis, wound-healing, bone metabolism, and diabetic disorders.

## Characteristics of WNT4

**Fig. 1:**
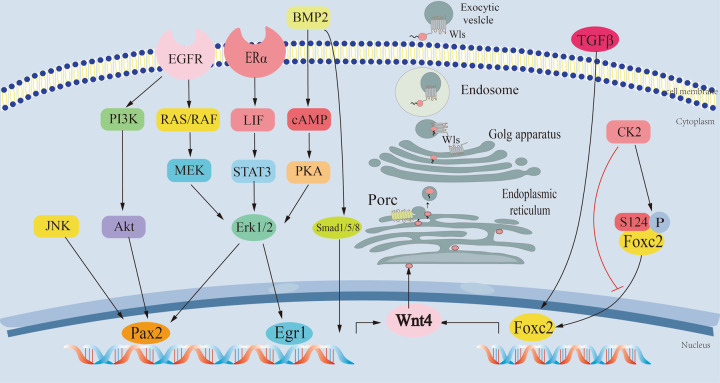
The upstream regulator of WNT4 and its transport mechanism in cells. EGR1, PAX2, and FOXC2 are three verified transcription factors that bind to the WNT4 promoter to increase its transcription. The reported upstream signaling pathways include ERα-LIF-STAT3-ERK1-EGR1, BMP2-cAMP-PKA-ERK1, RAS-RAF-MEK1/2-ERK1-EGR1, PI3K-AKT-PAX2, ERK1-PAX2, JNK-PAX2, TGFβ-FOXC2, and CK2/p-FOXC2. The cysteine-rich protein WNT4 can be modified with palmitoleic acid at Ser 212 by porcupine in the endoplasmic reticulum, where it is chaperoned to the cell membrane and secreted by interacting with WNTless/Evi (Wls).

WNT4 plays an important role in cell–cell interaction by encoding Cys-rich secretory proteins composed of 351 amino acids with 24 Cys residues conserved among other WNT family members [11]. WNT4 is a gly-protein with Asn 88 and Asn 297 as the possible asparagine-linked glycosylation sites. It contains one or more covalently linked carbohydrates of various types from monosaccharides to branch polysaccharides, including glycosylphosphatidylinositol and glycosaminoglycans. Glycosylation is necessary for WNT4 secretion. Knowing WNT4 is a secreted gly-protein, one question is: how WNT4 ligand is transported outside cells? Accumulating evidence indicates three different mechanisms, including transport by lipoprotein particles, exosomes, or autocrine. Just like other WNT ligands, the cysteine-rich protein WNT4 can be modified with palmitoleic acid at Ser 212 by porcupine in the endoplasmic reticulum [12], where it is chaperoned to the cell membrane and secreted by interacting with WNTless/Evi [1, 13]. Once secreted, the WNT protein signals in a paracrine manner and binds with the nearby receptor complex. Palmitoylation of WNT4 is required for its binding to its receptor frizzled (Fz1), but this is not essential for its secretion (Fig. 1). Other researchers [14] found that WNT4 secretion was PORCN-independent, and the secreted WNT4 did not exhibit paracrine activity but had a cell-autonomous activity, which indirectly proves the necessity of WNT4 palmitoylation.

The current validated upstream transcription factors of WNT4 include early growth response 1 (EGR1) [15], paired box 2 (PAX2) [16], and forkhead box C2 (FOXC2) [17, 18]. These transcription factors can bind with the WNT4 promoter and promote its transcription (Fig. 1). EGR1 is an important transcription factor, which participates in cell proliferation, differentiation, invasion, and apoptosis [19]. It is widely expressed in many cell types and can be activated through the mitogen-activated protein kinase (MAPK) signaling pathway, mainly depending on the RAS-RAF-MEK1/2-ERK1/2 signal transduction pathway. The activated EGR1 can either cause or inhibit the expression of its target genes, thus playing a part in transcriptional regulation [20]. EGR1 is significantly enriched in the WNT4 promoter during decidualization to regulate WNT4 expression [15]. When EGR1 is knocked down, the level of WNT4 is decreased significantly. This study also found that estrogen could drive WNT4 expression through estrogen receptor α–leukemia inhibitory factor–signal transducer and activator of transcription 3–EGR1 pathway in the mouse uterus [15]. Another study [12] further demonstrated that extracellular regulated protein kinases (ERK1) could promote the binding of EGR1 and PAX2 to the promoter region of WNT4 during osteogenic differentiation of bone marrow stromal cells (BMSCs). PAX2 also can activate the WNT4 promoter, which is the requisite for the development of the central nervous system, eye, ear, kidney, and mammary gland [21, 22]. The expression and activation of PAX2 is regulated by many signals, such as Akt, c-Jun N-terminal kinase (JNK), and ERK1/2 [22]. A key function of PAX2 is to activate WNT4 gene expression in metanephric mesenchymal cells during the development of renal tubules [23]. FOXC2 is a winged-helix/forkhead transcription factor and plays a crucial role in modulating cell proliferation, angiogenesis, and lymphangiogenesis [24]. Transforming growth factor (TGF)-β1 can promote its expression while casein kinase 2 (CK2) can inhibit translocation of endogenous FOXC2 to the nucleus through phosphorylating FOXC2 at the serine 124 site [25]. Studies [13, 14] showed that FOXC2 could also mediate osteogenic differentiation by binding with the WNT4 promoter in BMSCs. In myoblast cells, FOXC2 induced WNT signaling by directly interacting with the WNT4 promoter region, resulting in the elevated expression of bone morphogenetic protein-4 (BMP4) and RhoA-GTP proteins to suppress myogenesis, thus altering their lineage commitment toward osteogenesis [17]. Besides, WNT4 serves as a downstream effector of progesterone signaling, which is directly regulated by progesterone [26]. WNT4 is also necessary for BMP2 in decidualization, which promotes WNT4 expression through the Smad1/5/8-mediated signaling pathway [27]. Another study [24] found that BMP2 promoted WNT4 expression also through cAMP-protein kinase A-WNT4 signaling pathway during uterine decidualization. WNT4 is also the transcriptional target of Forkhead box O1 (FoxO1) [27], in which FoxO1 interacts with the progesterone receptor to coordinate cell cycle regulation and differentiation of human endometrial stromal cells (hESCs) [28]. However, there is so far no study reporting the binding of FoxO1 with the WNT4 gene promoter.

**Fig. 2:**
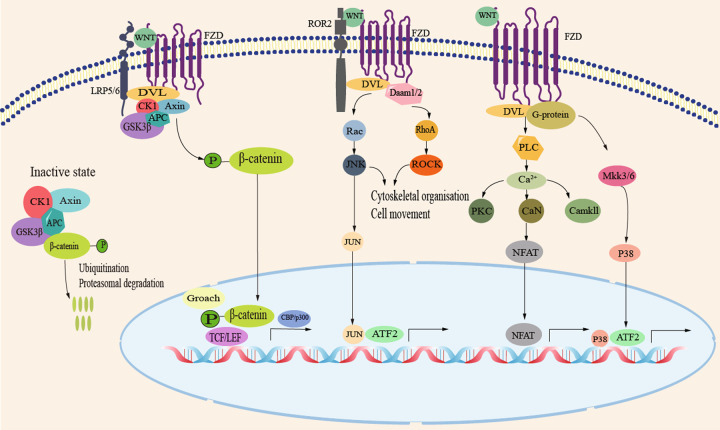
WNT4 signaling pathway. WNT4 regulates at least three distinct intracellular signaling pathways: β-catenin, planar cell polarity (PCP)/JNK, and Ca^2+^/CaMK pathways. In canonical WNT pathway, extracellular WNT ligand binds to FZD and LRP5/6 on the cell membrane and recruits DVL protein to promote dissociation of the β-catenin destruction complex, followed by accumulation of β-catenin, which translocates to the nucleus to interact with target downstream signaling molecules and activate intracellular signaling cascades. In WNT/PCP/JNK pathway, WNT4 binds to FZD and interacts between DVL and Dvl-associated activator of morphogenesis (DAAM) to activate the small GTPases Rac1 and Ras homolog gene family member A (RHOA). Rac1 activates JNK and phosphorylates c-Jun. In WNT/Ca^2+^ pathway, WNT4 ligand binds to FZD2 receptor and G protein subunits at the cell membrane and recruits DVL protein to promote PLC, leading to open calcium channels for Ca^2+^ release, resulting in intracellular Ca2+ accumulation. Ca^2+^ can activate the phosphates calcineurin (CaN) and several calcium-dependent kinases, including PKC and calmodulin-mediated kinase II (CaMKII). The phosphates calcineurin, in turn, activates the nuclear factor of activated T cells (NFAT) to regulate downstream cascades. The G protein-induced activation of p38 via mitogen-activated protein kinase 3/6 (MAPK 3/6) results in phosphorylation of activating transcription factor 2 (ATF2) on Thr69 and Thr71 to nuclear transcription.

The WNT protein can interact with >15 receptors, including Frizzled (FZD) receptors with ten isoforms and other co-receptors such as lipoprotein receptor-related protein (LRP), receptor tyrosine kinase-like orphan receptor, receptor-like tyrosine kinase, and protein tyrosine kinase 7 [1]. FZD receptors have typical G protein-coupled receptor features with seven membrane-spanning domains and extracellular N-terminal and hydrophilic C-terminal domains. WNT4 has been shown to regulate either β-catenin-dependent or β-catenin-independent WNT signaling based on the cell type manner (Fig. 2 and Table 1). For the WNT/β-catenin (also known as canonical signaling) pathway, β-catenin can be degraded by a complex of proteins including APC, Axin, glycogen synthase kinase (GSK)-3β, CK1, and DVL through phosphorylation and ubiquitination in the absence of WNT signal. Binding of the WNT ligand with its receptors (FZD and LRP) can induce the association of Axin with phosphorylated LRP. As a result, the destruction complex falls apart, and β-catenin is stabilized, which is subsequently transported to the nucleus where it binds with T cell factor to upregulate target genes. A study [16] showed that WNT4 could activate β-catenin through combining with FZD complexes (FZD1-LRP5 and FZD6-LRP6), thus promoting osteogenic differentiation of BMSCs. β-Catenin-independent (also known as non-canonical signaling) pathway, including the planar cell polarity (PCP) pathway, can stimulate cytoskeletal reorganization and the WNT-Ca^2+^ pathway, leading to calcium mobilization. For WNT/PCP/JNK pathway, WNT4 binds to FZD and interacts between DVL and Dvl-associated activator of morphogenesis to activate the small GTPases Rac1 and Ras homolog gene family member A. Rac1 activates JNK, which phosphorylates c-Jun. For WNT/Ca^2+^ pathway, WNT4 ligand binds to FZD2 receptor and G protein subunits at the cell membrane and recruits DVL protein to promote PLC, leading to open calcium channels for Ca^2+^ release, resulting in intracellular Ca^2+^ accumulation. Ca^2+^ can activate the phosphates calcineurin (CaN) and several calcium-dependent kinases, including PKC and calmodulin-mediated kinase II (CaMKII). The phosphates CaN, in turn, activates the nuclear factor of activated T cells to regulate downstream cascades. The G protein can induce activation of p38 via mitogen-activated protein kinase 3/6 (MKK3/6), resulting in phosphorylation of activating transcription factor 2 on Thr69 and Thr71 to nuclear transcription [29]. WNT4 can bind with Fzd2 to mediate the activation of the p38-JNK signaling pathway and improve bone formation [16]. In cervical cancer cells, the non-canonical WNT4/PCP/JNK pathway is engaged in promoting cell proliferation [30]. Meanwhile, another study [28] found that exogenous recombinant WNT4 protein could induce an influx of Ca^2+^ and cause phosphorylation of CaMKII in metanephric mesenchyme cells, thus inhibiting its proliferation.

**Table 1:** Summary of the expression and function of WNT4 in tissues of different diseases.

Disease	Cells	WNT4 expression	Effector(s)	Signaling	Consequence	References
Osteoporosis	BMSC	↓	BMP	WNT4/FZD2/p38-JNK	Osteogenic differentiation	[18]
			ERK1	WNT4/FZD5-6/β-catenin		[16]
			FOXC2			
			PTH			
Type 2 diabetes	β-cell	↑		WNT4/PCP/JNK	Promote β-cell maturation	[40]
				WNT4/ β-catenin	Inhibit β-cell proliferation and insulin secretion	[45]
Preeclampsia	Endometrial stromal cells	↓	BMP2	WNT4/ β-catenin	Improve decidualization	[51]
				WNT4/CaMKIIα		
Recurrent implantation failure		↓		WNT4/ β-catenin	Repair decidualization progress	[53]
Chronic kidney disease	Principal cells	↑		WNT4/ β-catenin	Promote myofibroblast transition	[66, 89]
Skin wound	Epithelial cells and fibroblast-like cells	↑	TGF-β	WNT4/ β-catenin	Improve wound healing	[78, 79]
Hyperprophic scars	Fibroblast cells	↓		WNT4/ERK	Inhibit α-SMA and Col 1 expression	[83]
Breast cancer	Breast cancer stem cell	↑	ER	WNT4/β-catenin	Proliferation	[90]
			PR	WNT4/PCP/JNK	Pro-carcinogenic	
Colorectal cancer	Colorectal cancer cell	↑		WNT4/β-catenin	Epithelial-to-mesenchymal transition	[7]
Laryngeal carcinoma	Laryngeal carcinoma cell	↑	PRMT5	WNT4/β-catenin	Induce laryngeal carcinoma cell proliferation, migration, and invasive capacity	[95]

## Role of WNT4 in cell differentiation

**Fig. 3:**
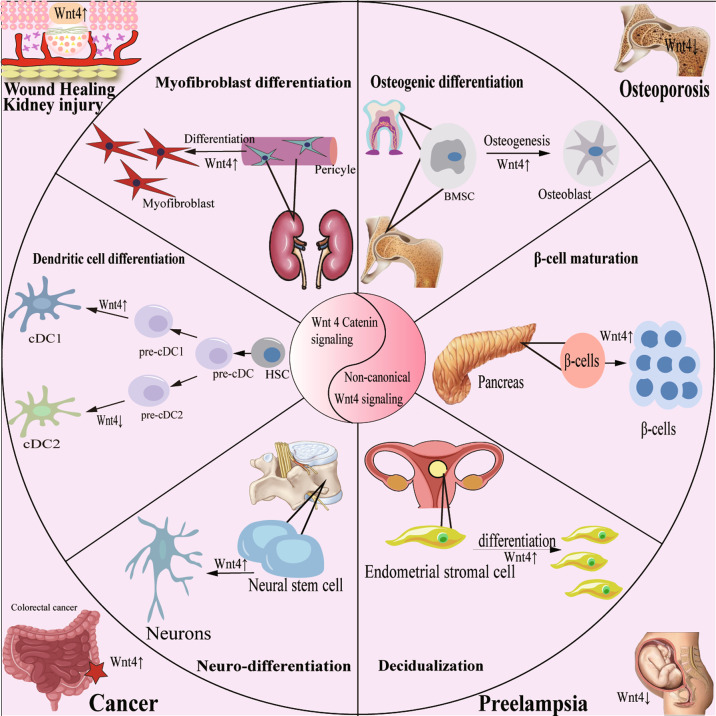
Function of WNT4 in cell differentiation and human diseases. WNT4 is a promoter of osteogenic differentiation, β-cell mutation, decidual cell decidualization, neuro-differentiation, DC differentiation, and myofibroblast differentiation and participates in the occurrence and progression of various diseases, including osteoporosis, preeclampsia, wound healing, kidney injury, and cancer.

Cell differentiation is a process in which a generic cell type develops into a specific cell type in response to specific triggers from the body or the cell itself. In addition to the critical role in embryonic development, cell differentiation also plays roles in various organisms, especially complex mammals, throughout their lives. Adult stem cell is a class of cells with multiple differentiation potentials existing in multiple areas of the body. It is currently known that WNT4 plays a role in mediating cell differentiation, including osteogenic differentiation, β-cell mutation, decidual cell decidualization, neuro-differentiation, dendritic cell (DC) differentiation, and myofibroblast differentiation and participates in the occurrence and progression of various diseases (Fig. 3).

### Role of WNT4 in osteogenic differentiation

Osteoblasts are important cells in the bone tissue, playing a key role in bone formation and maintenance of bone mass. BMSCs are the natural source for bone regeneration and can differentiate into osteoblasts. Then osteoblasts undergo three differentiation steps, including division growth, ground substance maturation, and mineralization. WNT4 protein is increased during differentiation of BMSCs to osteoblasts [31, 32]. WNT4 may play an important role in the crosstalk between BMP2 and RUNX1 [33]. WNT4 is also essential for melatonin-induced bone formation in the inflammatory environment both in human BMSCs and MC3T3-E1 pre-osteoblasts [16], in which ERK1/2-PAX2-EGR1 pathway was found to promote WNT4 transcription and expression. It was reported [18] that the transcription factor FOXC2 could transcriptionally regulate WNT4 expression during osteogenic differentiation of BMSCs. An early study [32] demonstrated that WNT4 could enhance osteogenic differentiation of BMSCs in vitro through activating p38 MAPK but not β-catenin pathway. But some recent target-specific small interfering RNA experiments [16] demonstrated that WNT4 could mediate the p38-JNK signaling pathway through interacting with Fzd2 and activate the β-catenin signaling pathway through interacting with Fzd1-LRP5 and Fzd6-LRP6 to induce osteogenesis. According to the latest view, WNT4 induces osteogenesis through both β-catenin and p38-JNK signaling pathways.

Human dental pulp stem cell (DPSC) is an autologous promising seed cell for bone tissue engineering. It was reported [34] that loss of WNT4 expression in DPSC inhibited odontogenic differentiation while overexpression of WNT4 in lipopolysaccharide-treated DPSC promoted odontogenic differentiation through JNK1 signaling pathways. WNT4 can also elevate osteogenic potential of stem cells from inflammatory dental pulp tissues for treating bone defects in rats [35]. WNT4 also plays a key role in promoting the osteogenesis process of adipose-derived stem cells (ADSCs) [36], which can be induced to differentiate into osteoblasts to mineralize extracellular matrix (ECM) and express proteins associated with bone phenotypes [37]. In ADSCs, parathyroid hormone (PTH) promotes the osteogenesis process through enhancing the expression of WNT4 [36].

### Role of WNT4 in β-cell maturation

Islet dysfunction and destruction lead to the hypofunction of insulin secretion, which is the common cause for both type 1 and type 2 diabetes mellitus (T2DM). The islets of Langerhans are highly vascularized miniorgans, so improving the mutation of islet β-cells and preserving the structural integrity and full function of the microvascular endothelium are two strategies for protecting the islets against inflammatory and oxidative stress assaults. Studies have shown that WNT4 is the most abundantly expressed WNT ligand in β-cells and its expression is enhanced during the postnatal functional maturation of human (mouse) islets. Its expression is enhanced by the high-fat high-sucrose diet [38–40]. Activating the non-canonical WNT4/PCP/JNK pathway has been demonstrated to induce β-cell maturation and increase glucose-stimulated insulin secretion (GSIS) [40] and increase the expression level of NKX6.1 and PDX1 in the human EndoC-β H1 β-cell line as well as human islets. Recent studies [39, 41] have suggested a therapeutic promise of human islet-like organoids from induced pluripotent stem cells for insulin-dependent diabetes, in which the non-canonical WNT4 pathway plays a major role in islet maturation through the induction of an ERRγ gene network to drive metabolic maturation of β-like cells. Some other studies [42] demonstrated that mesenchymal stromal cell (MSC)-based WNT4 therapies could promote angiogenesis of the islet by activating β-catenin signaling, thus promoting the survival and improve the endothelial function of the injured islet endothelium while WNT5a seemed to play a destructive role, suggesting that WNT4 may play a positive role in promoting islet maturation and protecting the function of the islet endothelium.

But in maturated pancreatic β-cell, the effect of WNT4 alone did not affect insulin secretion of primary murine islets [43], while knockdown of WNT4 inhibited cell proliferation and GSIS in β-cell line MIN6 [38]. WNT4 was found to be most abundantly expressed in β-cells and upregulated in the islets of two different insulin-resistant mouse strains [44] and islets of T2DM patients [45]. In addition, it could completely block canonical WNT3a signaling-stimulated cell growth and insulin secretion in INS-1 cells [46]. WNT3a functions as a canonical WNT activator to enhance β-cell proliferation and increase insulin secretion by activating the β-catenin/T cell-specific transcription factor 7-like 2 pathway, while WNT4 works as a canonical WNT inhibitor. Studies [45] discovered that WNT4 secretion was decreased while WNT3a secretion was increased in the pre-diabetic phase, and vice versa in the severe diabetic phase. Based on these findings, we speculate that WNT4 is necessary for β-cell differentiation, maturation, and function maintenance under physiological conditions, but under the pathological condition, it may work as a balancer with other WNT ligands such as WNT3a to regulate β-cell proliferation and insulin secretion. One reason is that WNT4 may work as a biphasic initiator for canonical and non-canonical WNT pathways, while non-canonical WNT/PCP signaling can inhibit the β-catenin pathway [47]. Under certain conditions, WNT4 actives the non-canonical PCP pathway to inhibit β-catenin activation.

### Role of WNT4 in DC decidual cell differentiation and decidualization

The differentiation of ESCs into decidual cells is a prerequisite for successful embryonic implantation and uterine decidualization. Increasing evidence indicates that impaired decidualization is the primary cause of early miscarriage in women and recurrent implantation failure in assisted reproduction [48, 49]. WNT4 is a critical regulator of embryo implantation and decidualization [50]. WNT4 deficiency is likely involved in the occurrence of preeclampsia [51]. WNT4-like protein is a cortical granule component to regulate preimplantation embryogenesis in mouse oocytes [52]. WNT4 gene variants are associated with the gestation duration [39]. Clinical research has demonstrated that WNT4 signaling is significantly overexpressed in the uterus during pregnancy, but inhibition of WNT4/β-catenin signaling interferes with the process of embryo implantation. Second, WNT4 protein in the decidual tissues of women with severe preeclampsia is significantly lower than that in normal pregnant women. Some in vitro studies [50] showed that knockdown of WNT4 in hESCs reduced the transcription of the decidualization markers, insulin-like growth factor-binding protein1 and prolactin.

Some factors can interfere with WNT4 protein to influence decidualization. The C-terminal Eps15 homology domain-containing 1 (EHD1), a critical repressor of the steroid hormone induction of decidualization, was found highly expressed in the endometrium of patients with repeated implantation failures [53]. EHD1 can interact with WNT4 to suppress WNT4/β-catenin signaling and impair decidualization in the endometrium [53]. Exposure to excessive norepinephrine significantly restricted the induced expression of the decidualized markers such as decidual prolactin-related protein, BMP2, WNT4, and heart and neural crest derivative-expressed transcript 2 in mice [54]. WNT4 can interact with FZD-2 to promote CaMKIIα signaling cascades calmodulin and calcineurin, thus attenuating the NF-κB transcriptional promoter activity in endometrial epithelial cells [55]. At the same time, microtubule depolymerization can inhibit WNT4 and FZD-2 interaction, thereby suppressing the downstream WNT4/CaMKIIα signaling cascades calmodulin and calcineurin, leading to implantation failure. WNT4 is also a downstream target protein of BMP2 to active β-catenin in the nucleus to control ESC decidualization [27]. The inhibition of WNT4/β-catenin signaling interferes with the process of embryo implantation [56]. So, WNT4 can activate not only β-catenin but also non-catenin pathways to promote differentiation of ESCs into decidual cells during the progress of decidualization.

### Role of WNT4 in neuro-differentiation

WNT4 can significantly promote the differentiation of neural stem cells (NSCs) into neurons by activating both β-catenin and MAPK/JNK pathways as well as suppressing the activation of Notch signaling [57]. In addition, transplantation of WNT4-modified NSC efficaciously repaired the injured spinal cord and recovered the motor function of hind limbs after spinal cord injury as shown in an in vivo study [57]. In amniotic epithelial cells, melatonin cooperated with WNT4 could increase bovine amniotic epithelial cell vitality and promote their differentiation into neural cells, which could be satisfactorily colonized into the injured spinal cord [58].

### Role of WNT4 in DC differentiation

DCs originate from hematopoietic stem cells (HSCs) and develop into progenitor cells. In the bone marrow, DC progenitor can differentiate into three types: plasmacytoid DCs, classical DCs (cDCs), and monocyte-derived DCs. Of them, cDCs are the most professional antigen-presenting cells of the immune system and can be dived into cDC1 and cDC2 based on the selective expression of cell surface molecules [59, 60]. cDC1 functions as an autonomous platform capable of antigen processing and priming for both CD4+ and CD8+ T cells and of the direct orchestration of their crosstalk that is required for optimal antitumor immunity. WNT4 overexpression in HSCs increased FLT^+^ DC differentiation [61] and increased progenitor frequency through activating the non-canonical PCP-JNK pathway [62], but not β-catenin-dependent canonical pathway. Recent research also found that WNT4 is a critical regulator of cDC1 vs. cDC2 development in mice [63]. WNT4 activating phosphoJNK/c-Jun pathway was required for pre-cDC1/cDC1 differentiation, while loss of WNT4 in CD11cCreWNT4flox/flox mice impaired differentiation of CD24+, Clec9A+, CD103+ cDC1 compared with CD11cCre controls. Conversely, lack of DC-derived WNT4 increased the cDC2 number and accelerated type 2 immunity marked by enhanced group 2 innate lymphoid cell (ILC) expansion, interleukin-5 production, and host resistance to the hookworm parasite *Nippostrongylus brasiliensis*. It appears that WNT4 could improve classical DC differentiation into cDC1 in responses to immunity against tumors, viruses, or microbes.

### Role of WNT4 in myofibroblast differentiation

Myofibroblasts are prototypical fibrotic cells involved in various pathological conditions, including liver, kidney, or lung fibrosis. In the kidney, myofibroblasts often appear de novo and accumulate in the interstitial space in response to injury, after which they synthesize excessive quantity of ECM to destruct the normal parenchymal tissue within solid organs in many chronic kidney diseases (CKD). Rene pericytes are the major source of scar-forming myofibroblasts during progressive CKD [64]. Rene pericytes are mesenchymal-derived cells embedded in the capillary basement membrane and direct contact with endothelial cells [65]. It functions to maintain microvascular stability and regeneration and regulate the cortical and medullary flow. WNT4 is one of the major accelerators of pericyte-to-myofibroblast trans-differentiation (PMT) [66]. During renal fibrosis, interstitial myofibroblasts, but not the tubular epithelium (except for the collecting duct), strongly express WNT4. WNT4 protein and its downstream genes β-catenin and Axin were increased in the adenine nephropathy rats [67]. Exogenous WNT4/β-catenin pathway could drive PMT through autocrine signaling [66], thus inducing the expression of α-smooth muscle actin (α-SMA) and differentiation of myofibroblasts in pericyte-like 10T1/2 mesenchymal cell line. But deletion of WNT4 did not modify myofibroblast differentiation during fibrosis. The reason is that β-catenin stabilization in interstitial stromal cells drives myofibroblast differentiation, while other WNT ligands expressed in the injured kidney also may compensate for the absence of WNT4. But the effect of WNT4 with non-canonical pathway on PMT has not been reported.

As the myofibroblasts function as elemental emergency cells when tissue or parenchymal repair is needed, myofibroblast differentiation is also appended when the lung and liver is injured. It was found [67] that both WNT4 and WNT5a levels were increased in the senile lung, which increased myofibroblast-like differentiation. The WNT signaling pathway is involved in the pathogenesis of liver fibrosis. In culture-activated HSCs, the expression of both canonical (β-catenin) and non-canonical (WNT4 and WNT5a) WNT genes were increased by approximately 3–12-fold compared with quiescent HSCs [68], demonstrating that the WNT signaling pathway contributes to HSC activation, leading to excessive ECM deposition.

## WNT4 and disease

### WNT4 and bone disease

The bone is a dynamic organ with constant remodeling throughout life. The maintenance of bone mineral density is dependent on the balance between bone resorption and bone formation, which is mediated by osteoclasts and osteoblasts, respectively. Clinical genome-wide association studies showed that WNT4 gene was associated with fracture risk [4], involving the lumbar spine [69], femoral, and total hip bone mineral density [70]. An in vivo study [71] showed that WNT4 attenuated bone loss in osteoporosis and skeletal aging mouse models by promoting bone formation and inhibiting bone resorption. Transgenic mice overexpressing WNT4 from osteoblasts demonstrated a protective effect of WNT4 against bone loss and chronic inflammation by inhibiting osteoclastogenesis and bone resorption [72]. Mechanistically, WNT4 inhibited NF-κB activation mediated by TGF-β-activated kinase-1 in macrophages and osteoclast precursors independent of β-catenin. In a study performed in our laboratory, we focused on the regulation of WNT4 on bone metabolism. The gene expression profile demonstrated that WNT4, WNT3a, and WNT5a were significantly downregulated in the bone tissue of patients with primary osteoporosis [73]. A recent study [74] reported that curculigoside, the active anti-osteoporosis compound extracted from *Curculigo orchioides* Gaertn, could targetedly activate ERK1 and then regulate the WNT4 expression in MC3T3-E1 cells (data under submission). Another in vivo study [32] showed that MSCs genetically engineered to express WNT4 could enhance osteogenesis and improve the repair of craniofacial defects in two different models of craniofacial bone injury, suggesting that WNT4 protein may prove to be a potential target for osteoporosis treatment.

### Role of WNT4 in wound healing and hypertrophic scar formation

Wound healing is a dynamic and synchronized process involving inflammation, cell proliferation and migration, ECM deposition, and remodeling. Numerous individual WNT ligands have been implicated in wound healing and the transition from fetal scarless to adult scar-forming repair [75]. WNT4 is expressed in early wounds in response to the provisional fibrin matrix [76]. It is located in regenerating epithelial cells and fibroblast-like cells beneath the epithelium after 2 h to 5 days postwounding [77]. TGF-β can directly increase the WNT4 expression in fetal and postnatal fibroblasts [78]. Some studies have proved that WNT4 is beneficial to wound healing [79–81]. MSC exosomes play an important role in tissue injury repair, in which WNT4 activates β-catenin signaling pathway to enhance proliferation and migration of skin cells [79]. Meanwhile, WNT4 also induces β-catenin activation in endothelial cells and exerts a proangiogenic effect [80], suggesting that exosome-delivered WNT4 may provide a new promise for the treatment of MSCs in cutaneous wound healing. An amniotic AlphaPatche containing concentrated molecules of PGE2, WNT4, and GDF-11 was demonstrated to be able to heal an otherwise non-healing wound successfully and completely in a 78-year-old male [81].

The hypertrophic scar tissue, which typically occurs after severe burn and traumatic injury of the skin, is a fibrotic disorder of cutaneous wound healing. Once the skin is injured, fibroblasts cells undergo a phenotype change into myofibroblasts, which increases EMC protein synthesis and contractile activity through upregulating α-SMA to facilitate wound healing and tissue repair. When the normal wound healing process is completed, myofibroblasts are removed via apoptosis. However, excessive synthesis and deposition of collagen proteins by myofibroblasts contribute to hypertrophic scar formation. It was found that the WNT4 expression in the fetal mouse skin was higher than that in the postnatal mouse skin, and the WNT4-null skin exhibited dermal fibroplasias, suggesting that WNT4 may play an anti-fibrotic role. The WNT4 protein expression was increased in the early stage of wound healing in postnatal mice, suggesting that WNT4 may play a pro-fibrotic role. But WNT4 expression was decreased in the hypertrophic scar tissue and fibroblasts derived from the scar tissue. TGF-β plays an important role in fibroblast activation and contractile activity [82]. WNT4 can inhibit the autocrine expression of TGF-β and reverse the phenotype change of fibroblasts through blocking the phosphorylation of Smad3 and ERK induced by TGF-β1. Meanwhile, WNT4 can downregulate the excessive expression levels of α-SMA and Col I in hypertrophic scar-derived fibroblasts [83]. So, in different physiological and pathological conditions, the expression and function of WNT4 are variant to present different functions. The main reason may be due to the activation of non-canonical WNT/PCP signaling by WNT4, thus inhibiting the β-catenin pathway, which is important for scar formation and excessive activation in hypertrophic scars [82].

### Role of WNT4 in Kidney injury

WNT4 was found to be significantly upregulated during the early phase of cisplatin-induced acute kidney injury (AKI) [84] and following ischemia–reperfusion injury in mice [85]. Clinical studies [82] found that increased urinary WNT4 expression was detected earlier than serum creatinine and epidermal growth factor receptor in patients with contrast-induced AKI after vascular intervention [85], suggesting that urinary WNT4 could potentially serve as a noninvasive biomarker for monitoring patients with tubular injury. WNT4 is known as a biomarker for monitoring patients with tubular injury, but some evidence suggests that increased WNT4 protein may contribute to the development of renal lesions in the course of kidney glomerular diseases. First, WNT4 expression was increased with the occurrence of renal fibrosis and progression of CKD [85, 86]. Renal and urinary WNT4 was unregulated in both ischemia–reperfusion injury and early phases of the cisplatin-induced AKI model [84, 85, 87]. Second, interstitial myofibroblasts located in the medulla but not in the cortical myofibroblasts or epithelium are the main source of WNT4 expression in kidney injury models. WNT4 was found to be expressed only in principal cells of papillary CDs and urothelium of kidney under basal conditions [66]. Under pathologic conditions, glomerular mesangial cells were activated, leading to hyperproliferation and myofibroblast-like cell differentiation [88]. The re-expressed WNT4 could increase rat glomerular mesangial cell growth in vitro through suppressing GSK-3β and improving β-catenin activity, while knocking down the WNT4 expression during experimental diabetes significantly promoted mesangial cell apoptosis [89]. In addition, WNT4 could improve PMT to increase kidney fibrosis and epithelial-to-mesenchymal (EMT) re-creation. So, inhibiting the WNT4 expression may be a beneficial strategy for kidney injury treatment.

### Role of WNT4 in cancers

WNT4 plays a pro-carcinogenic role in numerous cancer types. Research has shown that WNT4 is highly expressed in the human breast cancer tissue [90], thymoma tissue [91], and colorectal cancer (CRC) tissue [7]. The WNT4 level was elevated in the serum of CRC patients, and WNT4 expression was increased in the CRC tissue through releasing WNT4-rich exosomes [7]. Other studies also found that WNT4-rich exosomes were the main release route in CRC cells under hypoxia, which could be delivered to normoxic cells to enhance prometastatic behaviors [92]. High expression of WNT4 plays an important role in the development and progression of these cancers by improving the cancer cell proliferation, migration, and invasion capacities. Research has demonstrated that WNT4 could promote breast cancer stem cell proliferation [93] and the progression of gastric cancer [94]. In addition, WNT4 contributes to EMT and activated fibroblasts by activating the WNT4/β-catenin pathway in CRC tissues and cells [7]. So far as the action mechanism is concerned, estrogen and progesterone are the upstream agitators of WNT4 expression in mammary cancer. Both the WNT4/β-catenin and non-canonical pathway participate in tumor development based on the cell types. The oncogenic role of protein arginine methyltransferase 5 (PRMT5) could activate the WNT4/β-catenin signaling pathway to induce laryngeal carcinoma cell proliferation, migration, and invasion in vitro, as well as lymph node metastasis in vivo [95], while silencing WNT4 could reverse the effect of PRMT5. Human papillomavirus-induced E6 protein can increase WNT4 to activate the non-canonical WNT4/PCP/JNK pathway to promote cell proliferation in vitro and tumor growth in vivo [30]. Another study showed that the activation of JNK pathway by WNT4 overexpression was mediated in the development of thymoma [96]. WNT4 signaling is required for ILC cell proliferation and survival through regulating S6 kinase/mammalian target of rapamycin signaling to affect mitochondrial function and cellular metabolism [97]. So, inhibition of the WNT4 pathway is a possible treatment target of cancer disease.

## Conclusions and perspectives

Accumulating evidence indicates that WNT4 plays a major role in cell differentiation along with the process of human growth and development. It is reasonable to suppose that WNT4 and its signaling pathway may become molecular targets for disease diagnosis and treatment. Numerous studies have verified that both canonical and non-canonical WNT4 signaling pathways are associated with the progression of cell differentiation, indicating that all these WNT4 signaling pathways should be explored in future research. The summarized upstream pathways of WNT4, such as EGR1, PAX2, and FOXC1 transcript factors, provide novel therapeutic targets to treat WNT4-related diseases. In particular, the role of WNT4 as a promoter of osteogenic differentiation, decidualization, and myofibroblast differentiation makes it an attractive target for the development of drugs aimed at the therapy of osteoporosis, pregnancy, and wound healing. On the contrary, higher expression of WNT4 protein may lead to renal, hepatic, and lung fibrosis. One problem is whether a higher expression of WNT4 induced by tumors can promote hepatic, renal, and pulmonary fibrosis? Another contradiction to be solved is the different functions of WNT4 on the same cell at different stages. WNT4 is beneficial for β-cell maturation, but in maturated pancreatic β-cell, it does not affect insulin secretion. Also, in the same myofibroblasts, WNT4 promotes α-SMA secretion in myofibroblasts in the wound healing stage, while it inhibits α-SMA secretion in the hyperplasia scar stage. Although we hypothesize that WNT4 can initiate canonical or non-canonical pathways based on different conditions, more experiments are required to verify our hypothesis. For the treatment application, WNT4 protein has been successfully tried in wound healing, nerve repair, bone mineral density improvement, and assisted reproduction. However, its safety evaluation on the whole body needs further investigation.

## Data Availability

All data generated or analyzed during this study are included in this published article.

## References

[1] Nusse R, Clevers H (2017). Wnt/beta-catenin signaling, disease, and emerging therapeutic modalities. Cell.

[2] Boyer A, Goff AK, Boerboom D (2010). WNT signaling in ovarian follicle biology and tumorigenesis. Trends Endocrinol Metab.

[3] Eggers S, Ohnesorg T, Sinclair A (2014). Genetic regulation of mammalian gonad development. Nat Rev Endocrinol.

[4] Hendrickx G, Boudin E, Steenackers E, Nielsen TL, Andersen M, Brixen K (2017). Genetic screening of WNT4 and WNT5B in two populations with deviating bone mineral densities. Calcif Tissue Int.

[5] Mafra F, Catto M, Bianco B, Barbosa CP, Christofolini D (2015). Association of WNT4 polymorphisms with endometriosis in infertile patients. J Assist Reprod Genet.

[6] Zhang J, Zhang P, Shen Y, Yang M, Zou H, Liu H (2018). Relationship of WNT4 gene with the risk of epithelial ovarian cancer: a Han Chinese population-based association study. Genet Test Mol Biomarkers.

[7] Yang D, Li Q, Shang R, Yao L, Wu L, Zhang M (2020). WNT4 secreted by tumor tissues promotes tumor progression in colorectal cancer by activation of the Wnt/beta-catenin signalling pathway. J Exp Clin Cancer Res.

[8] Nicol B, Guerin A, Fostier A, Guiguen Y (2012). Ovary-predominant wnt4 expression during gonadal differentiation is not conserved in the rainbow trout (*Oncorhynchus mykiss*). Mol Reprod Dev.

[9] Kiewisz J, Skowronska A, Winiarska A, Pawlowska A, Kiezun J, Rozicka A (2019). WNT4 expression in primary and secondary kidney diseases: dependence on staging. Kidney Blood Press Res.

[10] Buckland J (2014). Osteoimmunology: dual role for Wnt4: bone formation and bone resorption. Nat Rev Rheumatol.

[11] Tanda N, Kawakami Y, Saito T, Noji S, Nohno T (1995). Cloning and characterization of Wnt-4 and Wnt-11 cDNAs from chick embryo. DNA Seq.

[12] Rios-Esteves J, Resh MD (2013). Stearoyl CoA desaturase is required to produce active, lipid-modified Wnt proteins. Cell Rep.

[13] Banziger C, Soldini D, Schutt C, Zipperlen P, Hausmann G, Basler K (2006). Wntless, a conserved membrane protein dedicated to the secretion of Wnt proteins from signaling cells. Cell.

[14] Rao DM, Shackleford MT, Bordeaux EK, Sottnik JL, Ferguson RL, Yamamoto TM (2019). Wnt family member 4 (WNT4) and WNT3A activate cell-autonomous Wnt signaling independent of porcupine O-acyltransferase or Wnt secretion. J Biol Chem.

[15] Liang XH, Deng WB, Li M, Zhao ZA, Wang TS, Feng XH (2014). Egr1 protein acts downstream of estrogen-leukemia inhibitory factor (LIF)-STAT3 pathway and plays a role during implantation through targeting Wnt4. J Biol Chem.

[16] Li X, Li Z, Wang J, Li Z, Cui H, Dai G (2019). Wnt4 signaling mediates protective effects of melatonin on new bone formation in an inflammatory environment. FASEB J.

[17] Gozo MC, Aspuria PJ, Cheon DJ, Walts AE, Berel D, Miura N (2013). Foxc2 induces Wnt4 and Bmp4 expression during muscle regeneration and osteogenesis. Cell Death Differ.

[18] Zhou P, Li Y, Di R, Yang Y, Meng S, Song F (2019). H19 and Foxc2 synergistically promotes osteogenic differentiation of BMSCs via Wnt-beta-catenin pathway. J Cell Physiol.

[19] Wang B, Guo H, Yu H, Chen Y, Xu H, Zhao G (2021). The role of the transcription factor EGR1 in cancer. Front Oncol.

[20] Kim JH, Jeong IY, Lim Y, Lee YH, Shin SY (2011). Estrogen receptor beta stimulates Egr-1 transcription via MEK1/Erk/Elk-1 cascade in C6 glioma cells. BMB Rep.

[21] Panneerselvam A, Kannan A, Mariajoseph-Antony LF, Prahalathan C (2019). PAX proteins and their role in pancreas. Diabetes Res Clin Pract.

[22] Zhou TB (2012). Signaling pathways of PAX2 and its role in renal interstitial fibrosis and glomerulosclerosis. J Recept Signal Transduct Res.

[23] Torban E, Dziarmaga A, Iglesias D, Chu LL, Vassilieva T, Little M (2006). PAX2 activates WNT4 expression during mammalian kidney development. J Biol Chem.

[24] Kume T (2008). Foxc2 transcription factor: a newly described regulator of angiogenesis. Trends Cardiovasc Med.

[25] Golden D, Cantley LG (2015). Casein kinase 2 prevents mesenchymal transformation by maintaining Foxc2 in the cytoplasm. Oncogene.

[26] Brisken C, Heineman A, Chavarria T, Elenbaas B, Tan J, Dey SK (2000). Essential function of Wnt-4 in mammary gland development downstream of progesterone signaling. Genes Dev.

[27] Li Q, Kannan A, Das A, Demayo FJ, Hornsby PJ, Young SL (2013). WNT4 acts downstream of BMP2 and functions via beta-catenin signaling pathway to regulate human endometrial stromal cell differentiation. Endocrinology.

[28] Takano M, Lu Z, Goto T, Fusi L, Higham J, Francis J (2007). Transcriptional cross talk between the forkhead transcription factor forkhead box O1A and the progesterone receptor coordinates cell cycle regulation and differentiation in human endometrial stromal cells. Mol Endocrinol.

[29] Xu X, Zhang M, Xu F, Jiang S (2020). Wnt signaling in breast cancer: biological mechanisms, challenges and opportunities. Mol Cancer.

[30] Zhao L, Wang L, Zhang C, Liu Z, Piao Y, Yan J (2019). E6-induced selective translation of WNT4 and JIP2 promotes the progression of cervical cancer via a noncanonical WNT signaling pathway. Signal Transduct Target Ther.

[31] Leitao L, Neto E, Conceicao F, Monteiro A, Couto M, Alves CJ (2020). Osteoblasts are inherently programmed to repel sensory innervation. Bone Res.

[32] Chang J, Sonoyama W, Wang Z, Jin Q, Zhang C, Krebsbach PH (2007). Noncanonical Wnt-4 signaling enhances bone regeneration of mesenchymal stem cells in craniofacial defects through activation of p38 MAPK. J Biol Chem.

[33] Zhang HL, Yang ZQ, Duan CC, Geng S, Wang K, Yu HF (2018). WNT4 acts downstream of BMP2 to mediate the regulation of ATRA signaling on RUNX1 expression: implications for terminal differentiation of antler chondrocytes. J Cell Physiol.

[34] Zhong TY, Zhang ZC, Gao YN, Lu Z, Qiao H, Zhou H (2019). Loss of Wnt4 expression inhibits the odontogenic potential of dental pulp stem cells through JNK signaling in pulpitis. Am J Transl Res.

[35] Zhong T, Gao Y, Qiao H, Zhou H, Liu Y (2020). Elevated osteogenic potential of stem cells from inflammatory dental pulp tissues by Wnt4 overexpression for treating bone defect in rats. Ann Palliat Med.

[36] An Y, Zhao J, Nie F, Wu Y, Xia Y, Li D (2019). Parathyroid hormone (PTH) promotes ADSC osteogenesis by regulating SIK2 and Wnt4. Biochem Biophys Res Commun.

[37] Halvorsen YD, Franklin D, Bond AL, Hitt DC, Auchter C, Boskey AL (2001). Extracellular matrix mineralization and osteoblast gene expression by human adipose tissue-derived stromal cells. Tissue Eng.

[38] Kurita Y, Ohki T, Soejima E, Yuan X, Kakino S, Wada N (2019). A high-fat/high-sucrose diet induces WNT4 expression in mouse pancreatic beta-cells. Kurum Med J.

[39] Yoshihara E, O’Connor C, Gasser E, Wei Z, Oh TG, Tseng TW (2020). Immune-evasive human islet-like organoids ameliorate diabetes. Nature.

[40] Bader E, Migliorini A, Gegg M, Moruzzi N, Gerdes J, Roscioni SS (2016). Identification of proliferative and mature beta-cells in the islets of Langerhans. Nature.

[41] Yoshihara E, Wei Z, Lin CS, Fang S, Ahmadian M, Kida Y (2016). ERRγ is required for the metabolic maturation of therapeutically functional glucose-responsive β cells. Cell Metab.

[42] Wang L, Qing L, Liu H, Liu N, Qiao J, Cui C (2017). Mesenchymal stromal cells ameliorate oxidative stress-induced islet endothelium apoptosis and functional impairment via Wnt4-beta-catenin signaling. Stem Cell Res Ther.

[43] Heller C, Kühn MC, Mülders-Opgenoorth B, Schott M, Willenberg HS, Scherbaum WA (2011). Exendin-4 upregulates the expression of Wnt-4, a novel regulator of pancreatic β-cell proliferation. Am J Physiol Endocrinol Metab.

[44] Krützfeldt J, Stoffel M (2010). Regulation of wingless-type MMTV integration site family (WNT) signalling in pancreatic islets from wild-type and obese mice. Diabetologia.

[45] Lee SH, Demeterco C, Geron I, Abrahamsson A, Levine F, Itkin-Ansari P (2008). Islet specific Wnt activation in human type II diabetes. Exp Diabetes Res.

[46] Bowen A, Kos K, Whatmore J, Richardson S, Welters HJ (2016). Wnt4 antagonises Wnt3a mediated increases in growth and glucose stimulated insulin secretion in the pancreatic beta-cell line, INS-1. Biochem Biophys Res Commun.

[47] Mentink RA, Rella L, Radaszkiewicz TW, Gybel T, Betist MC, Bryja V (2018). The planar cell polarity protein VANG-1/Vangl negatively regulates Wnt/β-catenin signaling through a Dvl dependent mechanism. PLoS Genet.

[48] Wang X, Yu Q (2018). An update on the progress of transcriptomic profiles of human endometrial receptivity. Biol Reprod.

[49] Koot YE, Teklenburg G, Salker MS, Brosens JJ, Macklon NS (2012). Molecular aspects of implantation failure. Biochim Biophys Acta.

[50] Franco HL, Dai D, Lee KY, Rubel CA, Roop D, Boerboom D (2011). WNT4 is a key regulator of normal postnatal uterine development and progesterone signaling during embryo implantation and decidualization in the mouse. FASEB J.

[51] Wang G, Zhang Z, Chen C, Zhang Y, Zhang C (2016). Dysfunction of WNT4/WNT5A in deciduas: possible relevance to the pathogenesis of preeclampsia. J Hypertens.

[52] Liu M, Yang HT (2016). WNT4-like protein is a cortical granule component in mouse oocytes and functions in regulating preimplantation embryogenesis. Syst Biol Reprod Med.

[53] Zhou Q, Yan G, Ding L, Liu J, Yu X, Kong S (2019). EHD1 impairs decidualization by regulating the Wnt4/beta-catenin signaling pathway in recurrent implantation failure. EBioMedicine.

[54] Wang J, Tang Y, Wang S, Cui L, Li D, Du M (2021). Norepinephrine exposure restrains endometrial decidualization during early pregnancy. J Endocrinol.

[55] Shukla V, Kaushal JB, Kumar R, Popli P, Agnihotri PK, Mitra K (2019). Microtubule depolymerization attenuates WNT4/CaMKIIalpha signaling in mouse uterus and leads to implantation failure. Reproduction.

[56] Mohamed OA, Jonnaert M, Labelle-Dumais C, Kuroda K, Clarke HJ, Dufort D (2005). Uterine Wnt/beta-catenin signaling is required for implantation. Proc Natl Acad Sci USA.

[57] Li X, Peng Z, Long L, Tuo Y, Wang L, Zhao X (2020). Wnt4-modified NSC transplantation promotes functional recovery after spinal cord injury. FASEB J.

[58] Gao Y, Bai C, Zheng D, Li C, Zhang W, Li M (2016). Combination of melatonin and Wnt-4 promotes neural cell differentiation in bovine amniotic epithelial cells and recovery from spinal cord injury. J Pineal Res.

[59] Mildner A, Jung S (2014). Development and function of dendritic cell subsets. Immunity.

[60] Murphy TL, Grajales-Reyes GE, Wu X, Tussiwand R, Briseno CG, Iwata A (2016). Transcriptional control of dendritic cell development. Annu Rev Immunol.

[61] Louis I, Heinonen KM, Chagraoui J, Vainio S, Sauvageau G, Perreault C (2008). The signaling protein Wnt4 enhances thymopoiesis and expands multipotent hematopoietic progenitors through beta-catenin-independent signaling. Immunity.

[62] Heinonen KM, Vanegas JR, Lew D, Krosl J, Perreault C (2011). Wnt4 enhances murine hematopoietic progenitor cell expansion through a planar cell polarity-like pathway. PLoS ONE.

[63] Hung LY, Johnson JL, Ji Y, Christian DA, Herbine KR, Pastore CF (2019). Cell-intrinsic Wnt4 influences conventional dendritic cell fate determination to suppress type 2 immunity. J Immunol.

[64] Kuppe C, Kramann R (2016). Role of mesenchymal stem cells in kidney injury and fibrosis. Curr Opin Nephrol Hypertens.

[65] Duffield JS (2014). Cellular and molecular mechanisms in kidney fibrosis. J Clin Investig.

[66] DiRocco DP, Kobayashi A, Taketo MM, McMahon AP, Humphreys BD (2013). Wnt4/beta-catenin signaling in medullary kidney myofibroblasts. J Am Soc Nephrol.

[67] La L, Wang L, Qin F, Jiang J, He S, Wang C (2018). Zhen-wu-tang ameliorates adenine-induced chronic renal failure in rats: regulation of the canonical Wnt4/beta-catenin signaling in the kidneys. J Ethnopharmacol.

[68] Kovacs T, Csongei V, Feller D, Ernszt D, Smuk G, Sarosi V (2014). Alteration in the Wnt microenvironment directly regulates molecular events leading to pulmonary senescence. Aging Cell.

[69] Estrada K, Styrkarsdottir U, Evangelou E, Hsu YH, Duncan EL, Ntzani EE (2012). Genome-wide meta-analysis identifies 56 bone mineral density loci and reveals 14 loci associated with risk of fracture. Nat Genet.

[70] Styrkarsdottir U, Halldorsson BV, Gretarsdottir S, Gudbjartsson DF, Walters GB, Ingvarsson T (2008). Multiple genetic loci for bone mineral density and fractures. N Engl J Med.

[71] Yu B, Chang J, Liu Y, Li J, Kevork K, Al-Hezaimi K (2014). Wnt4 signaling prevents skeletal aging and inflammation by inhibiting nuclear factor-kappaB. Nat Med.

[72] Yu B, Chang J, Liu Y, Li J, Kevork K, Al-Hezaimi K (2015). Addendum: Wnt4 signaling prevents skeletal aging and inflammation by inhibiting nuclear factor-kappaB. Nat Med.

[73] SQ L, EY F, BY X, LH X, J C, HJ X (2017). Study on the gene expression profile in the bone tissue in primary osteoporosis with kidney yang deficiency syndrome. Chin J Osteopros.

[74] Zhang Q, Zhao L, Shen Y, He Y, Cheng G, Yin M (2019). Curculigoside protects against excess-iron-induced bone loss by attenuating Akt-FoxO1-dependent oxidative damage to mice and osteoblastic MC3T3-E1 cells. Oxid Med Cell Longev.

[75] Burgy O, Konigshoff M (2018). The WNT signaling pathways in wound healing and fibrosis. Matrix Biol.

[76] Labus MB, Stirk CM, Thompson WD, Melvin WT (1998). Expression of Wnt genes in early wound healing. Wound Repair Regen.

[77] Okuse T, Chiba T, Katsuumi I, Imai K (2005). Differential expression and localization of WNTs in an animal model of skin wound healing. Wound Repair Regen.

[78] Colwell AS, Krummel TM, Longaker MT, Lorenz HP (2006). Wnt-4 expression is increased in fibroblasts after TGF-beta1 stimulation and during fetal and postnatal wound repair. Plast Reconstr Surg.

[79] Zhang B, Wang M, Gong A, Zhang X, Wu X, Zhu Y (2015). HucMSC-exosome mediated-Wnt4 signaling is required for cutaneous wound healing. Stem Cells.

[80] Zhang B, Wu X, Zhang X, Sun Y, Yan Y, Shi H (2015). Human umbilical cord mesenchymal stem cell exosomes enhance angiogenesis through the Wnt4/beta-catenin pathway. Stem Cells Transl Med.

[81] Riordan NH, George BA, Chandler TB, McKenna RW (2015). Case report of non-healing surgical wound treated with dehydrated human amniotic membrane. J Transl Med.

[82] Chawla S, Ghosh S (2018). Regulation of fibrotic changes by the synergistic effects of cytokines, dimensionality and matrix: Towards the development of an in vitro human dermal hypertrophic scar model. Acta Biomater.

[83] Liu J, Zhao B, Zhu H, Pan Q, Cai M, Bai X (2020). Wnt4 negatively regulates the TGF-beta1-induced human dermal fibroblast-to-myofibroblast transition via targeting Smad3 and ERK. Cell Tissue Res.

[84] He YX, Diao TT, Song SM, Wang CC, Wang Y, Zhou CL (2018). Wnt4 is significantly upregulated during the early phases of cisplatin-induced acute kidney injury. Sci Rep.

[85] Zhao SL, Wei SY, Wang YX, Diao TT, Li JS, He YX (2016). Wnt4 is a novel biomarker for the early detection of kidney tubular injury after ischemia/reperfusion injury. Sci Rep.

[86] Wei SY, Wang YX, Zhang QF, Zhao SL, Diao TT, Li JS (2017). Multiple mechanisms are involved in salt-sensitive hypertension-induced renal injury and interstitial fibrosis. Sci Rep.

[87] Ali RM, Al-Shorbagy MY, Helmy MW, El-Abhar HS (2018). Role of Wnt4/beta-catenin, Ang II/TGFbeta, ACE2, NF-kappaB, and IL-18 in attenuating renal ischemia/reperfusion-induced injury in rats treated with Vit D and pioglitazone. Eur J Pharmacol.

[88] Zhao JH (2019). Mesangial cells and renal fibrosis. Adv Exp Med Biol.

[89] Lin CL, Wang JY, Huang YT, Kuo YH, Surendran K, Wang FS (2006). Wnt/beta-catenin signaling modulates survival of high glucose-stressed mesangial cells. J Am Soc Nephrol.

[90] Vouyovitch CM, Perry JK, Liu DX, Bezin L, Vilain E, Diaz JJ (2016). WNT4 mediates the autocrine effects of growth hormone in mammary carcinoma cells. Endocr Relat Cancer.

[91] Chen Y, Liu X, Liu Y, Wang Y, Wang H, Lu C (2017). Decreased Wnt4 expression inhibits thymoma development through downregulation of FoxN1. J Thorac Dis.

[92] Huang Z, Yang M, Li Y, Yang F, Feng Y (2018). Exosomes derived from hypoxic colorectal cancer cells transfer Wnt4 to normoxic cells to elicit a prometastatic phenotype. Int J Biol Sci.

[93] Brisken C, Hess K, Jeitziner R (2015). Progesterone and overlooked endocrine pathways in breast cancer pathogenesis. Endocrinology.

[94] Zhu Y, Zhang B, Gong A, Fu H, Zhang X, Shi H (2016). Anti-cancer drug 3,3’-diindolylmethane activates Wnt4 signaling to enhance gastric cancer cell stemness and tumorigenesis. Oncotarget.

[95] Wang N, Yan H, Wu D, Zhao Z, Chen X, Long Q (2020). PRMT5/Wnt4 axis promotes lymph-node metastasis and proliferation of laryngeal carcinoma. Cell Death Dis.

[96] Chen Y, Zhang P, Tang P, Lv P, Li X, Wang Y (2018). Wnt4 overexpression promotes thymoma development through a JNK-mediated planar cell polarity-like pathway. Oncol Lett.

[97] Shackleford MT, Rao DM, Bordeaux EK, Hicks HM, Towers CG, Sottnik JL (2020). Estrogen regulation of mTOR signaling and mitochondrial function in invasive lobular carcinoma cell lines requires WNT4. Cancers.

